# Suppression of cancer stemness p21-regulating mRNA and microRNA signatures in recurrent ovarian cancer patient samples

**DOI:** 10.1186/1757-2215-5-2

**Published:** 2012-01-19

**Authors:** Michael F Gallagher, Cynthia CBB Heffron, Alexandros Laios, Sharon A O'Toole, Brendan Ffrench, Paul C Smyth, Richard J Flavin, Salah A Elbaruni, Cathy D Spillane, Cara M Martin, Orla M Sheils, John J O'Leary

**Affiliations:** 1 Department of Histopathology, University of Dublin, Trinity College. Trinity Centre for Health Sciences, St James' Hospital, Dublin 8, Ireland; 2 Molecular Pathology Research Laboratory, Coombe Women's and Infants' University Hospital, Dublin 8, Ireland; 3 The Department of Obstetrics and Gynecology, Trinity Centre for Health Sciences, St James' Hospital, Dublin 8, Ireland; 4 The Department of Histopathology, Cork University Hospital, Wilton, Cork, Ireland

**Keywords:** Ovarian cancer, chemoresistance, recurrent disease, cancer stem cell, p53, p21

## Abstract

**Background:**

Malignant ovarian disease is characterised by high rates of mortality due to high rates of recurrent chemoresistant disease. Anecdotal evidence indicates this may be due to chemoresistant properties of cancer stem cells (CSCs). However, our understanding of the role of CSCs in recurrent ovarian disease remains sparse. In this study we used gene microarrays and meta-analysis of our previously published microRNA (miRNA) data to assess the involvement of cancer stemness signatures in recurrent ovarian disease.

**Methods:**

Microarray analysis was used to characterise early regulation events in an embryonal carcinoma (EC) model of cancer stemness. This was then compared to our previously published microarray data from a study of primary versus recurrent ovarian disease. In parallel, meta-analysis was used to identify cancer stemness miRNA signatures in tumor patient samples.

**Results:**

Microarray analysis demonstrated a 90% difference between gene expression events involved in early regulation of differentiation in murine EC (mEC) and embryonic stem (mES) cells. This contrasts the known parallels between mEC and mES cells in the undifferentiated and well-differentiated states. Genelist comparisons identified a cancer stemness signature set of genes in primary versus recurrent data, a subset of which are known p53-p21 regulators. This signature is present in primary and recurrent or in primary alone but essentially never in recurrent tumors specifically. Meta-analysis of miRNA expression showed a much stronger cancer stemness signature within tumor samples. This miRNA signature again related to p53-p21 regulation and was expressed prominently in recurrent tumors. Our data indicate that the regulation of p53-p21 in ovarian cancer involves, at least partially, a cancer stemness component.

**Conclusion:**

We present a p53-p21 cancer stemness signature model for ovarian cancer. We propose that this may, at least partially, differentially regulate the p53-p21 mechanism in ovarian disease. Targeting CSCs within ovarian cancer represents a potential therapeutic avenue.

## Background

Ovarian cancer is the leading gynecological malignancy, affecting more than 200,000 women per annum worldwide [1,2]. This is largely due to high rates of chemoresistant recurrence associated with the disease. Primary ovarian cancer develops silently, with most patients symptom-free, only presenting at an advanced stage. Treatment of primary disease generally consists of surgical removal of the malignancy in combination with platinum-based treatments. In recent years, chemotherapeutic agent carboplatin has proved successful in eliminating primary malignancy while reducing side effects for the patient [Reviewed in [3]]. Mechanistically, platinum-based drugs bind nucleotides within the DNA backbone, causing cross-linking. In response, cells activate DNA-repair mechanisms that ultimately result in apoptosis. Today, the majority of primary ovarian malignancies are successfully treated, where up to 80% of women will recover [2]. The remaining 20% may be explained by late presentation of the disease by asymptomatic women. Alarmingly, up to 80% of these survivors will develop chemoresistant terminal recurrent disease within two years, which is accepted as the main factor in fatality rates. We have previously used comparative microarray analysis to demonstrate that primary and recurrent disease have substantially different gene and microRNA (miRNA) expression profiles [4,5], which we continue in this study.

Current treatment of recurrent disease, which is similar to treatment of primary disease, has proved ineffective. Thus, recurrent disease must be fully characterised and novel therapeutic approaches developed. One such approach involves targeting cancer cells with stemness properties. These cancer stem cells (CSCs) have been described in ovarian cancer [Reviewed in [6]] and have several properties with relevance to recurrent ovarian cancer. CSCs are sufficient to regenerate malignancy *in vivo *via extensive self-renewal and differentiation. Tumor regeneration from CSCs is remarkably efficient, where a single CSC is often sufficient to re-establish disease [7,8]. CSCs proliferate well in the hypoxic conditions found in the tumor microenvironment [9,10]. As they differentiate, CSCs quickly develop neo-vasculature to fuel further tumorigenesis. Perhaps the most alarming aspect of CSCs is their uninhibited proliferation in the presence of chemotherapeutic agents. It is broadly accepted that CSCs play a role in most, if not all, primary malignancies. Theoretically, the persistence of a single CSC post-intervention could be sufficient to explain chemoresistant recurrence. However, the role of CSCs in recurrent ovarian disease is poorly understood. Ultimately we must develop methods of targeting specific CSC populations as part of a combined anti-cancer strategy.

Many studies have demonstrated the presence of CSCs in ovarian malignancy [6]. However, establishing ovarian CSC models in culture has proved challenging. In this study we employed an embryonal carcinoma (EC) model of cancer stemness. Originally derived from malignant teratomas that can develop in the ovary, EC cells are the original and best characterised CSC model [11-14]. We have previously shown high relevance between EC cells and ovarian serous carcinoma patient samples at the miRNA level [15]. Pluripotent EC cells can differentiate into cells representing all three germ layers and are considered the malignant equivalent of embryonic stem (ES) cells [11-14]. Nullipotent EC cells can avoid differentiation *in vivo *to generate poorly-differentiated, highly-malignant tumors [11-14]. Comparison of ES cells with pluripotent and nullipotent EC cells can establish mechanisms required for functional malignant differentiation. The cells are so similar that EC cells are used as an easily cultured model of ES biology, reflecting the difficulty of targeting CSCs without damaging non-malignant stem cell populations [16-18].

In this study we first used gene microarrays to assess upstream regulation of differentiation in murine EC (mEC) and mES cells. Our analysis describes aberrant regulation of differentiation in EC cells. Subsequently, we compared mEC genelists to our previously published primary versus recurrent tumor sample data [5]. We described the presence of a cancer stemness p53-p21 regulatory mechanism in ovarian tumor samples. This mechanism is employed by primary disease and suppressed in recurrent disease. Subsequently, we conducted a meta-analysis of our previously published human EC (hEC) and tumor sample miRNA data [15,6]. We report that cancer stemness signature miRNAs are more relevant to ovarian cancer than cancer stemness signature genes. We detail substantial recruitment of stemness signature miRNAs by recurrent disease. Thus recurrent tumors suppress and activate stemness signature genes and miRNAs respectively. Our analysis indicates that cancer stemness mechanisms are specifically and differentially regulated in primary and recurrent ovarian malignancy, with obvious implications for treatment.

## Methods

### Cell Culture

Murine ES (ES-E14TG2a) and EC cells (pluripotent 'SCC-PSA1' and nullipotent 'Nulli-SCC1') were purchased from ATCC, cultured on murine irradiated fibroblasts in DMEM supplemented with 10% foetal bovine serum, 4 mM L-glutamine (Invitrogen) and 100 U/ml of penicillin/streptomycin (Invitrogen Corporation, Carlsbad, CA, USA) and spontaneously differentiated via removal of feeder layer. Human EC cells were retinoic acid-differentiated as previously described [15].

### Tumor Samples

Tumor sample data was previously published [5,6]. Briefly, two cohorts of primary and recurrent samples were assessed. Cohort 1 contained 5 primary and recurrent serous papillary adenocarcinomas (Grade 3). Cohort 2 contained 3 paired ovarian cancers from the same patient but with different histologies: papillary serous, mixed mullerian and clear cell carcinomas.

### Microarray Analysis

RNA was isolated using the RNeasy kit (Qiagen, West Sussex, UK) as per manufacturer's protocol. Digoxigenin-UTP labelled cRNA was synthesized via the Chemiluminescent RT-IVT Labelling Kit v2.0 (Life Technologies, Foster City, CA, USA) and hybridized to Mouse Genome Survey arrays (Life Technologies) as per manufacturers' instructions. Data was filtered to a signal/noise ration threshold > 3 in at least one sample using R and further analysed using Spotfire^® ^(Life Technologies). Genelists were generated using cut-offs of 0.05 (p-Value) and ± 2.0 (fold change). Functional relationships were analysed using DAVID [19,20]. Pathways associations of predicted targets of miRNAs highlighted were generated using DIANA miRPath [21] using cut-offs of ≥ 2 genes per pathway and p-value ≤ 0.05.

### qPCR Analysis

2 μg total RNA was used to synthesis cDNA using the High Capacity cDNA Archive Kit (Life Technologies) as per manufacturer's instructions. Microarrays were validated using 36 pre-designed TaqMan assays (Life Technologies). Gene expression values were generated using the 2^-ddCt method [22]. microRNA was isolated using the mirVANA kit (Ambion) and miRNA TaqMan qPCR (Life Technologies) [23] analysis carried out as previously described [5,6,15]. Data plotted represents the mean value across a minimum of n = 3. Error bars represent standard error of the mean.

## Results

### Microarray analysis of early mEC and mES differentiation

It is well established that ES and EC cells express similar gene profiles in the undifferentiated and well-differentiated (one week or later) states [16,11,18]. In contrast, our understanding of the earlier, upstream regulation of differentiation is sparse. We hypothesized that comparison of early differentiation of mES and mEC cells would identify cancer-specific differences in upstream regulation of stem cell differentiation. Addressing this we used microarray analysis to assay early (three day) differentiation of mES and mEC cells.

**Figure 1:**
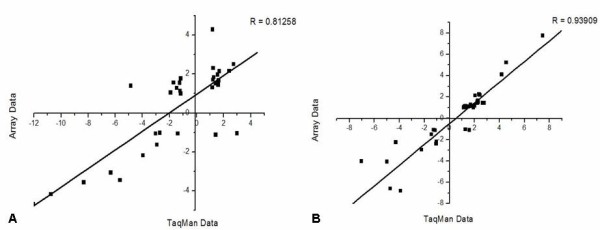
**Validation of mEC and mES microarray data**. Microarray data was validated through qPCR (TaqMan) analysis of a group of 36 genes. Data presented represents gene expression change in differentiated cells compared to undifferentiated and shows good correlation for mES (A) and PSA-SCC1 (B) datasets.

Microarray data was validated through qPCR analysis, showing excellent correlation (Figure 1). An overview of the number of differentially expressed genes in pluripotent (SCC-PSA1) and nullipotent (Nulli-SCC) mEC and mES cells is shown in Table 1. At cut-offs of 0.05 (p-Value) and ± 2.0 (fold change) SCC-PSA1 cells alter the expression of 724 genes: 202 upregulated and 522 downregulated at fold change levels between +18 and -18 (Additional File 1). Top ten SCC-PSA1 genes are characterised by receptor activity and growth and differentiation/development roles (Table 2). Noteworthy events include upregulation of apoptosis (Bcl)-related gene Bid3 and downregulation of Cav2 tumor suppressor [24] and metastasis-linked Nupr1 [25]. Functional relationship analysis identified upregulation of developmental pathways and downregulation of transcription regulation processes and Toll-Like Receptor (TLR), Interleukin-2 and cancer pathways (Additional File 1).

**Table 1:** An overview of the numbers and percentage overlap of differentially expressed genes (D/U) during early differentiation of mES and mEC cells.

Cell Type	Gene Number		% Overlap		
	**Upreg**	**Downreg**	**mES**	**SCC-PSA1**	**Nulli-SCC**

**mES**	554	832		10	< 1

**SCC-PSA1**	202	522	33		< 1

**Nulli-SCC**	185	152	< 1	< 1	

**Table 2:** Top ten genes differentially expressed (D/U) during early differentiation of mES and mEC cells.

Gene Symbol	GeneName	Fold Change
SCC-PSA1		
**Upregulated**		
Olfr1450	Olfactory receptor 1450	9.0
Fgf5	Fibroblast growth factor 5	8.9
Dscam	Down syndrome cell adhesion molecule	7.7
Afp	Alpha fetoprotein	5.2
Rbp4	Retinol binding protein 4, plasma	5.0
Slc28a2	Solute carrier family 28, member 2	4.5
Bid3	BH3 interacting domain, apoptosis agonist	4.3
Igfbp5	Insulin-like growth factor binding protein 5	4.2
Irs4	Insulin receptor substrate 4	3.8
		
**Downregulated**		
Clec2d	C-type lectin domain family 2, member d	-11.3
Fbln5	Fibulin 5	-6.5
Cav2	Caveolin 2	-5.6
Irf5	Interferon regulatory factor 5	-5.5
Lsp1	Lymphocyte specific 1	-5.1
Olfr787	Olfactory receptor 787	-4.9
Fxyd4	FXYD-containing ion transport regulator 4	-4.8
Nupr1	Nuclear protein 1	-4.7
Kcnk4	K channel, subfamily K, member 4	-4.6
Cpne2	Copine II	-4.6
**Nulli-SCC**		
**Upregulated**		
Flt1	FMS-like tyrosine kinase 1	5.3
Npy5r	Neuropeptide Y receptor Y5	5.0
Olfr786	Olfactory receptor 786	4.4
Fau	FBR-MuSV ubiquitously expressed	4.1
Gm392	Gene model 392, (NCBI)	3.7
Gm449	Gene model 449, (NCBI)	3.2
Loxl2	Lysyl oxidase-like 2	2.9
Aoc3	Amine oxidase, copper containing 3	2.8
Serpini2	Serine proteinase inhibitor 1	2.8
Olfr870	Olfactory receptor 870	2.7
		
**Downregulated**		
Ssa2	Sjogren syndrome antigen A2	-5.3
4930486G11Rik	RIKEN cDNA 4930486G11 gene	-4.6
1700052K11Rik	RIKEN cDNA 1700052K11 gene	-4.5
Refbp2	RNA and export factor binding protein 2	-4.4
2900011O08Rik	RIKEN cDNA 2900011O08 gene	-3.7
Tmem62	Transmembrane protein 62	-3.7
Tirap	TIR domain-containing adaptor protein	-3.6
Defb13	Defensin beta 13	-3.5
Es31	Esterase 31	-3.4
Nap1l5	Nucleosome assembly protein 1-like 5	-3.3
**mES**		
**Upregulated**		
H1foo	H1 histone family, member O, oocyte-specific	64.2
Mrgprh	MAS-related GPR, member H	60.8
Mak10	MAK10 homolog	59.6
V1rd11	Vomeronasal 1 receptor, D11	57.8
B230317F23Rik	RIKEN cDNA B230317F23 gene	41.1
Gdpd3	Glycerophosphodi- phosphodiesterase 3	39.2
Na	Gene model 979, (NCBI)	32.3
Eif2c4	Euk translation initiation factor 2C, 4	30.6
Tll1	Tolloid-like	22.9
Na	Similar to Ig gamma-2a chain precursor	20.1
**Downregulated**		
MP4	Proline rich protein MP4	-70
Pck1	Phosphoenolpyruvate carboxykinase 1	-49.3
Fgfrl1	Fibroblast growth factor receptor-like 1	-38.3
Rpgrip1l	Rpgrip1-like	-20.6
Olfr508	Olfactory receptor 508	-16.0
Eif5a2	Euk translation initiation factor 5A2	-15.6
9130015A21Rik	RIKEN cDNA 9130015A21 gene	-14.6
Tecta	Tectorin alpha	-11.0
Fancc	Fanconi anemia, complementation group C	-10.7
Pax9	Paired box gene 9	-9.9

Nulli-SCC cells responded to differentiation stimuli through the upregulation of 185 and downregulation of 152 genes at levels from -6.3 to 14.0 fold (Additional File 2). Top ten genes included signal transducers and regulators of development/differentiation and malignancy (Table 2). Notable genes include hypoxia and tumor growth regulator Loxl2 [26] and tumor suppressor Serpini2 [27]. Interestingly Ssa2 is downregulated, a gene that is commonly expressed on the surface of apoptotic cells. Functional analysis identified upregulation of signal transduction regulators and downregulation of growth regulators (Additional File 2).

Upstream differentiation of mES cells is characterized by substantial levels of upregulation: 554 upregulated and 832 downregulated genes at levels of 232 to -68 fold (Additional File 3). Top ten genes are populated with receptors and developmental regulators (Table 2). Tll1 is linked to cardiac development, the first organised system formed during embryogenesis. Notably, a key RNAi gene, Eif2c4, is upregulated during differentiation, perhaps reflective of involvement of the RISC complex [28]. Upregulated mES genes regulate development, signalling and gene expression while downregulated genes regulate morphogenesis, particularly growth factor binding. Stemness-linked pathways such as Wnt-catenin and Hedgehog signalling were upregulated while signalling pathways including TLR and TGF-ß were downregulated (Additional File 3).

### Aberrant upstream regulation of differentiation in mEC cells

A comparison of mES and mEC early differentiation genelists is summarised in Table 1 and detailed in additional files 1, 2 and 3. In contrast to documented undifferentiated and well-differentiated comparisons, 90% of the mES genelist differed to the mEC genelist at this earlier time point (Table 1). Similarly, almost 70% of the SCC-PSA1 genelist differed from the mES genelist (Table 1). Functional relationship analysis indicates that quite different mechanisms are activated during early differentiation of mEC and mES cells. This included mES-specific upregulation of p53 signaling pathway genes (Additional File 3). There is very little overlap between Nulli-SCC and the other cell types (Additional Files 1, 2 and 3). Only four genes are upregulated by SCC-PSA1 and downregulated by Nulli-SCC cells, while only two are downregulated by both cell types. The downregulation of symporters, signal-transducing membrane proteins, which are upregulated by pluripotent cells, may indicate a potential counteraction of differentiation. Upstream regulation of differentiation represents a substantial difference between these cell types, supporting our hypothesis. While similar genes maintain the self-renewal state in each cell, different mechanisms are employed to regulate the early events in differentiation.

### A SCC-PSA1 p53 mechanism is expressed in primary and maintained in recurrent tumors

**Figure 2:**
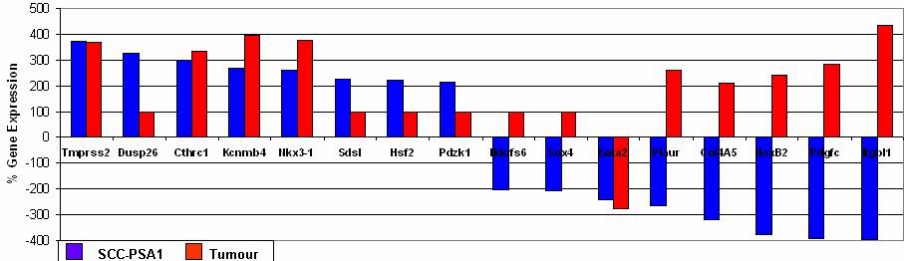
**Expression of SCC-PSA1 stemness signature mRNAs in recurrent tumors**. Sixteen genes were identified as differentially expressed in SCC-PSA1 mEC cells and in tumor data. Data is presented as the percentage change in gene expression in differentiated compared to undifferentiated SCC-PSA1 cells (blue) and in primary tumor samples compared to recurrent (red). Gene Expression values are detailed in table 3 (Microarray data p-Value ≤ 0.05).

We have previously published microarray analysis of primary versus recurrent tumor samples [5,6]. The study contained two cohorts. Cohort 1 represents a group of matched primary and recurrent tumors while cohort 2 represents primary and recurrent tumor samples from the same patients. In this study, raw microarray data from the primary versus recurrent study was reanalysed in an identical fashion to mES and mEC data described above (Additional File 4). Primary versus recurrent disease and mEC genelists were then compared. Genes altered similarly in mEC and mES data were not considered to be cancer-specific and were removed from this analysis. Comparison of mEC and tumor data identified 16 SCC-PSA1 genes expressed in tumor samples (Figure 2, Table 3). These genes group into those that are A) expressed in both primary and recurrent tumors and B) those expressed in primary but not recurrent tumors (Table 3). Many of these genes have links to stemness and malignancy. Tmprss2 is a transmembrane signalling protein that is upregulated in prostate cancer [29]. Cthrc1 is a Smad2/3 (TGF-β signalling) inhibiting Wnt signalling modulator that is differentially expressed in invasive breast cancer and several solid tumors [30]. Nkx3-1 is a metastatic marker transcription factor expressed in prostate cancer [31]. Pdgfc is a cisplatin-associated growth factor [32]. Col4a5 is linked to several cancers while Plaur is a regulator of tissue reorganisation [33]. Ndufs6 is an oxidative phosphorylation enzyme linked to cervical cancer [34]. Pdzk1 is linked to oestrogen-sensitivity in breast and ovarian cancers [35]. Sdsl is a cancer-specific metabolic enzyme [36]. Only one gene, Gata2, an endodermal differentiation marker [37] was upregulated by mEC cells and expressed higher in primary tumors than in recurrent.

**Table 3:** Percentage gene expression of mEC-specific genes expressed in primary versus recurrent tumor samples (Group A expressed similarly in primary and recurrent samples).

	% Gene Expression			% Gene Expression	
Gene Name	Tumor	SCC-PSA1	Gene Name	Tumor	Nulli-SCC
	(P/R)	(U/D)		(P/R)	(U/D)
**Group A**			**Group A**		
Dusp26		325.2	Egln3		259.9
Hsf2		222.5	Ndufab1		245.4
Pdzk1		212.8	Gpr6		229.6
Sdsl		226.2	Ltbr		212.2
Ndufs6		-203.1	Golga5		-208.5
Sox4		-208.1	Slc15a1		-214.6
Group B			Gpatc3		-236.7
Itgbl1	432.6	-396.1	Dgcr8		-323.3
Kcnmb4	397.0	267.5	Tirap		-364.3
Nkx3-1	375.2	260.0	Group B		
Tmprss2	368.3	373.0	Cask	442.1	-220.5
Cthrc1	334.6	298.7	Stau2	292.9	-201.6
Pdgfc	283.2	-392.8	Bnip3	283.4	217.4
Plaur	259.9	-263.2	Pfkp	239.4	251.0
Hoxb2	239.3	-375.5	Pak6	202.4	-213.8
Col4a5	210.8	-320.7			
Gata2	-276.5	-243.0			

When scrutinised, we noted that several of the genes highlighted above have been defined as p53 regulators in various models, as now described. Dusp26 is a p53-inhibiting phosphatase that negatively regulates proliferation of epithelial cells [38]. Stemness gene Sox4 is a p16 and p53 regulator in cancer cells [39] while Hsf2 is a regulator of p53 stability [40]. Hoxb2 has been linked to p205 regulation of p53 and is a well known regulator of EC differentiation [41]. Collectively, our analysis indicates that both primary and recurrent ovarian tumors express this 'p53-regulating stemness signature'.

### A NULLI-SCC p21 mechanism is suppressed by recurrent tumors

**Figure 3:**
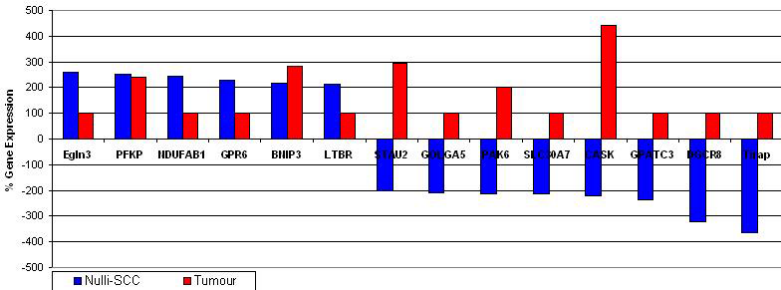
**Expression of Nulli-SCC stemness signature mRNAs in recurrent tumors**. Fourteen genes were identified as differentially expressed in Nulli-SCC mEC cells and in tumor data. Data is presented as the percentage change in gene expression in differentiation-stimulated compared to undifferentiated Nulli-SCC cells (blue) and in primary tumor samples compared to recurrent (red). Gene Expression values are detailed in table 3 (Microarray data p-Value ≤ 0.05).

Despite the reduced genelist size, 14 Nulli-SCC genes were expressed in A) both primary and recurrent tumors or B) primary tumors only (Figure 3, Table 3). These genes related to apoptosis/cellular proliferation, signaling and regulation. Dgcr8 (Pasha) is a key miRNA biosynthesis gene [42], while Tirap is a regulator of TLR signaling [43]. TNF-family related Ltbr and hypoxia-linked Egln3 are apoptosis regulators [44,45]. Gpr6 is a development regulator expressed in umbilical cord cells [46]. Ndufab1 is a TGF-β signaling related NADPH enzyme [47]. Slc15a1 is involved in drug absorption in the small intestine and has been linked to several cancers and metastasis [48]. Coupled with this is the recurrent suppression of apoptosis regulators Bnip3 and Stau2 [49,50]. Notably, two p21 regulators are expressed higher in primary tumors compared to recurrent: Cask mediates the expression of p21 to control cell proliferation [51] while Pak6 is a p21 interacting kinase that is a required for chemoresistance in prostate cells [52]. Collectively, an EC cancer stemness signature expressed in tumor samples is linked to maintained p53 regulation and suppression of p53's main target, p21, in recurrent disease.

### Recruitment of cancer stemness signature miRNAs during recurrence

**Figure 4:**
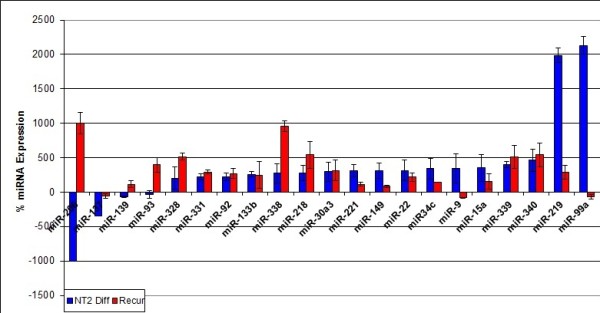
**Expression of NTera2 stemness signature miRNAs in recurrent tumors**. Twenty one miRNAs were identified as differentially expressed in differentiated NTera2 hEC cells and in tumor data. Data is presented as the percentage change in miRNA expression in differentiated NTera2 hEC cells (blue) and in recurrent tumors compared to primary (red). Values represent the mean of at least n = 3 and error bars the standard error of the mean.

**Figure 5:**
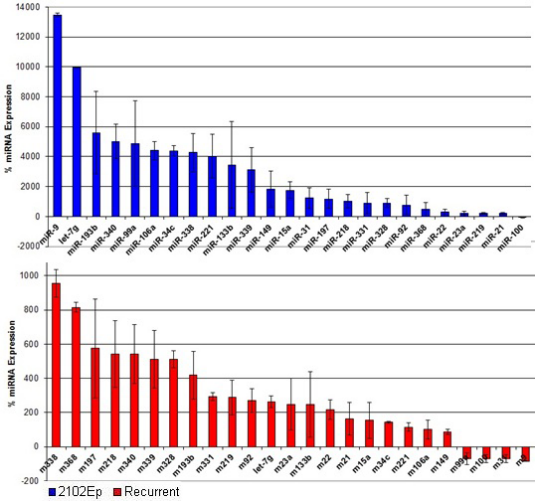
**Expression of 2102Ep stemness signature miRNAs in recurrent tumors**. Twenty six miRNAs were identified as differentially expressed in undifferentiated 2102Ep compared to undifferentiated NTera2 hEC cells and in tumor data. Data is presented as log_10 _(fold change). miRNAs presented showed altered expression in undifferentiated 2102Ep cells compared to undifferentiated NTera2 (blue) and in recurrent tumors compared to primary (red). Values represent the mean of at least n = 3 and error bars the standard error of the mean.

Having identified gene level overlaps, we next conducted overlap meta-analysis of our previously published miRNA data for primary and recurrent patient samples and human EC (hEC) early (three day) differentiation [5,6,15]. The earlier study identified cancer stemness signature miRNAs: those miRNAs involved in the differentiation of hEC cells. Specifically, our previous tumor study highlighted 60 miRNAs (52 up and 8 downregulated) in recurrent disease [6]. Of these, 55 miRNAs (92%) are expressed in hEC cells (Additional File 5). 21 recurrent disease-specific miRNAs are linked to differentiation of pluripotent NTera2 hEC cells (Figure 4). We have previously shown that nullipotent 2102Ep hEC cells express a large number of miRNAs at substantially higher levels than NTera2 cells [15]. Here we report that 26 (43%) recurrent disease-specific miRNAs are expressed at higher levels in 2102Ep cells than in NTera2 (Figure 5). Thus, development of recurrent tumors involves recruitment of cancer stemness signature miRNAs. Specific examples include miR-9, which is the most downregulated miRNA in recurrent tumors and is > 1000% higher expressed in undifferentiated 2102Ep cells compared to NTera2, and miR-206, which is in the top ten miRNAs upregulated by recurrent tumors and downregulated during NTera2 differentiation. Molecular pathway relationships between predicted gene targets of the miRNAs highlighted were identified using DIANAmirPATH (Additional file 5). While little pathway overlap was observed in gene array data, miRNA data showed strong pathway associations. Pathway analysis highlighted alteration of several cancer pathways (miRs-10b, -100, -106, -107, -128 and let-7g) as well as Wnt and TGF-β stemness signaling pathways (mirs-10b, -100, -106, -128a and 137). Finally, we assessed the expression of p53-p21 regulating miRNAs in these datasets. Two miRNAs, miRs-106a and b, are validated targets of p21 [52] that are upregulated in recurrent disease and expressed in hEC cells. Notably, miR-106b expression in 2102Ep cells is double that of NTera2 cells. In contrast, miR-155, the only validated p53-regulating miRNA, is unaltered in recurrent tumors. We note that the p53 signaling pathway was highlighted for let-7g and miRs-106b and -107 in pathway analysis (Additional file 5). In overview, we find that miRNAs linked to 2102Ep malignancy are highly relevant to primary and recurrent tumors.

## Discussion

Although CSCs are obvious suspects in the development of recurrent ovarian malignancy, a relationship has yet to be established or described in detail. Anecdotal evidence includes altered regulation of Notch3 in chemoresistant ovarian disease and the clear parallel between epithelial-mesenchymal transition (EMT) and CSC differentiation mechanisms [53,54]. In this study we conducted microarray and meta-analysis of mRNA and miRNA expression in primary and recurrent tumor samples and an EC model of cancer stemness. Our analysis reiterates that development of primary and recurrent ovarian disease involves quite different mechanisms: thousands of genes are differentially expressed. At the gene level, recurrent tumors appear to repress a cancer stemness signature related to p53-p21 regulation. In parallel, recurrent tumors recruit a population of miRNAs with close links to the development of highly malignant, poorly-differentiated tumors from nullipotent hEC cells.

**Figure 6:**
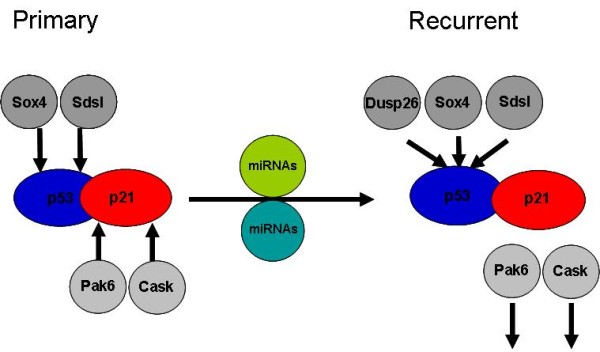
**Suppression of cancer stemness p53-p21 regulation in recurrent tumors**. Primary disease is characterised by the expression of p53-regulating stemness signature genes Sox4 and Sdsl, which is continued and enhanced with Dusp26 expression in the recurrency. In contrast, p21 regulating stemness signature genes Pak6 and Cask are expressed in primary disease and suppressed in recurrency. This process is paralleled by recruitment of stemness signature miRNAs by recurrent disease.

Different genetic profiles are employed by primary and recurrent ovarian tumors [5,6]. In this study we demonstrate that malignant stem cell differentiation genes are expressed in either primary tumors or both primary and recurrent tumors but essentially never in recurrent tumors-specifically. Some CSC mechanisms are similarly employed in primary and recurrent tumorigenesis. In addition, an obvious implication of our study is that CSCs that survive chemotherapy to repopulate recurrent disease can do so using different mechanisms than those employed in primary disease. Functional relationship analysis indicated that these stemness signature genes have a particular relevance to cellular proliferation and apoptosis. Several of the genes highlighted are known 'p53-p21 signaling regulators'. Mechanistically this relates to regulation of p53-p21 processes, where p53 regulation is enhanced and p21 regulation no longer required in recurrent tumors. This is supported by increased expression of p21 repressing miRNAs in recurrent tumors and strong predicted targeting of p53 signaling genes by tumor-specific miRNAs. Altered p53-p21 regulation is the primary mechanism through which cancers avoid apoptosis and stimulate cellular proliferation. Predictably, we did not find loss of p53 or p21 in recurrent disease (data not shown). It appears that p53-p21 regulation is required at both stages of ovarian malignancy. In Figure 6 we present a schematic to illustrate the p53-p21 regulators highlighted in out study. We propose that these genes and miRNAs regulate p53-p21 signaling, at least partially, in primary and recurrent disease (Figure 6). Indeed, this is likely to be a component of a larger mechanism. This p53-p21 regulating component appears to play a role in primary tumors that is not used during recurrence. We refer to this as a p53-p21 regulating mechanism within the cancer stemness signature (genes altered during differentiation of EC cells but not by ES cells). As a key tumorigenesis component, differential regulation of stemness-linked p53-p21 mechanisms in primary and recurrent disease is an important outcome of this study and will be the subject of ongoing analysis.

It is well established that EC and ES cells are highly similar in the undifferentiated and well-differentiated states [16-18]. This illustrates the significant challenges to the concept of targeting CSCs in a manner that does not harm the non-malignant stem cell pool. In this study we have identified upstream regulation of differentiation as a substantial difference between EC and ES cells, supporting our hypothesis. While downregulated mEC and mES genes displayed similarity, upregulated SCC-PSA1 genes were almost 90% specific to malignancy. This supports a model where normal and malignant stem cells employ similar mechanisms to maintain the self-renewal state. The different phenotypes developing from differentiation, therefore, are related to activation of specific malignant or non-malignant genes. Both cell types alter genes related to similar processes: receptor-mediated signalling of development/differentiation. Thus the differentiation of malignant and non-malignant cells is driven by a divergent group of genes. It is noteworthy that the primary-recurrent genetic switch contained an equally strong Nulli-SCC cell signature, despite the much reduced genelist. Nulli-SCC cells avoid differentiation through maintained levels of gene and miRNA expression to generate highly malignant tumors [11]. While a small number of molecular events take place in these cells response to differentiation, these appear to have a particular relevance to the difference between primary and recurrent disease. Stemness genes are never expressed by recurrent disease only, suggesting a less stem-like profile. These genes have a particular relevance to cellular proliferation and apoptosis, including p53-p21 regulation. Of particular note is the downregulation in Nulli-SCC cells of TLR signaling adapter Tirap, a gene that is constantly expressed in primary and recurrent disease. TLR signaling has received increased attention in both cancer and stemness studies in recent years [55]. In summary, recurrent disease appears to have more correlation with nullipotent cells rather than EC cells with good differential potential.

Recurrent tumor development involves the suppression of twice as many genes as are specifically activated (Cohort 1). This indicates that recurrent malignancy does not require a substantial number of mechanisms employed by primary tumors. Specifically, angiogenesis and development genes are turned off by recurrent disease as malignancy genes are turned on. The upregulation of polycystic ovary-associated gene Fabp4 and ovarian cancer gene Prkcbp1 may be of particular importance. There was little overlap between genes altered in cohort 1 and cohort 2, which altered genes more associated with malignancy and less with differentiation. Functional relationship analysis revealed that recurrent disease no longer requires homeostasis or stimulus response processes while upregulating catalytic activity and protein binding process. In general, recurrent disease behaves more as a developing cancer rather than the chemical stress responses required by primary disease.

## Conclusion

CSCs targeting is a potential avenue through which treatment of recurrent, chemoresistant ovarian cancer may be improved. This is complicated by the similarities between cancer and non-cancer stem cells and our poor understanding of recurrent ovarian disease. We have identified the early events of stem cell differentiation as a key area of difference between cancer and non-cancer stem cells. Furthermore, we have highlighted the association of a p53-p21 related cancer stemness signature within ovarian disease. Our data suggests that a stem cell involved in development of recurrent disease employs different mechanisms of tumorigenesis. Our study suggests that it may be possible to target early differentiation events in CSCs without damaging non-cancer stem cells, which would have broad implications for treatments. Our data indicates that such therapies should be independently tailored for primary and recurrent ovarian disease. CSC targeting during treatment of primary disease is likely to have a negative impact on recurrent tumorigenesis. CSC targeting in recurrent disease should be developed with consideration to independent mechanisms. Development of strategies to achieve this will continue in our group.

## Abbreviations

CSC: cancer stem cell; EC: embryonal carcinoma; h: human; mEC: murine embryonal carcinoma (cells); mES: murine embryonic stem (cells); miRNA: microRNA; P/R: Primary compared to recurrent; qPCR: quantitative polymerase chain reaction; U/D: Undifferentiated compared to differentiated

## Competing interests

The authors declare that they have no competing interests.

## Authors' contributions

MFG designed and carried out the comparisons and was the main author of the manuscript. CCBBH, MFG and PCS carried out microarray experiments and analysis. BF conducted statistical analysis. AL, SAO'T, RJF, SAE, CMM, OMS were involved in the original experiments and contributed to data analysis and interpretation. CDS contributed to manuscript design. JJO'L oversaw the project. All authors have read and approved the final manuscript.

## Supplementary Material

Additional file 1 **Microarray and functional relationship analysis of early differentiation response of SCC-PSA1 cells. **This file contains genelists of genes upregulated and downregulated by SCC-PSA1 cells in response to differentiation stimulus. This file also contains results of analysis to identify functional relationships between these genes. Click here for file

Additional file 2 **Microarray and functional relationship analysis of early differentiation response of NULLI-SCC cells. **This file contains genelists of genes upregulated and downregulated by NULLI-SCC cells in response to differentiation stimulus. This file also contains results of analysis to identify functional relationships between these genes. Click here for file

Additional file 3 **Microarray and functional relationship analysis of early differentiation response of mES cells. **This file contains genelists of genes upregulated and downregulated by mES cells in response to differentiation stimulus. This file also contains results of analysis to identify functional relationships between these genes. Click here for file

Additional file 4 **Microarray and functional relationship analysis of primary versus recurrent tumor samples. **This file contains genelists of genes upregulated and downregulated in primary versus recurrent tumor samples. This file also contains results of analysis to identify functional relationships between these genes. Click here for file

Additional file 5 **Comparison of microRNA expression in hEC and primary versus recurrent tumor samples. **This file details the relative expression patterns and levels of microRNAs in hEC cells and primary versus recurrent tumor samples and the pathway associations of genes targeted by these microRNAs. Click here for file
